# Concentric Split Aluminum with Silicon-Aluminum Nitride Annular Rings Resonators

**DOI:** 10.3390/mi10050296

**Published:** 2019-04-30

**Authors:** Muhammad Ammar Khan, Jing-Fu Bao, Fei-Hong Bao, Xin Zhou

**Affiliations:** School of Electronic Science and Engineering, University of Electronic Science and Technology of China, Chengdu 611731, China; imbaofh@std.uestc.edu.cn (F.-H.B.); xzhou@std.uestc.edu.cn (X.Z.)

**Keywords:** phononic crystal, bandgap, anchor loss, high quality factor, Silicon-Aluminum Nitride (Si-AlN) Micro-Electro-Mechanical-Systems (MEMS) resonator

## Abstract

This paper presents a novel approach of annular concentric split rings microelectromechanical resonators with tether configuration to reduce anchor loss and gives very high-quality factor (*Q*) 2.97 Million based on FEA (Finite Element Analysis) simulation. The operating frequencies of these resonators are 188.55 MHz to 188.62 MHz. When the proposed SR (square rectangle) hole shaped one dimensional phononic crystal (1D PnC), and two dimensional phononic crystal (2D PnC) structure consist of very wide and complete band gaps is applied to novel design rings MEMS resonators, the quality factor (*Q*) further improved to 19.7 Million and 1750 Million, respectively, by using the finite element method. It is also observed that band gaps become closer by reducing the value of filling fraction, and proposed SR PnC gives extensive peak attenuation. Moreover, harmonic response of ring resonator is verified by the perfect match layers (PML) technique surrounded by resonators with varying width 1.5*λ*, and 3*λ* effectively reduce the vibration displacement.

## 1. Introduction

Nowadays, Micro-Electro-Mechanical-Systems (MEMS) components have been widely used in the field of engineering science, and technology because of their high efficiency, miniature in size, low power consumption, and low cost fabrication [1,2,3,4,5,6,7].

Several researchers [8,9,10,11,12,13,14,15,16] have demonstrated the ring resonators or (disc resonator with hole is developed in center). It is noted that operating frequency, and design of resonator play an important role in the performance and improving the quality factor of resonators. Moreover, the phononic crystal (PnC) having wide acoustic band gap is the key factor that can give the high-quality *Q* of microelectromechanical resonators. 

To obtain the wide band gap, a lot of PnC structures have been designed [17,18,19,20,21,22,23,24,25], and applied to resonators of different shapes [18,19,20,22]. Phononic crystal can reduce the energy dissipated through anchors. Quality *Q* of the MEMS resonators has improved with phononic crystals extended to two dimension (2D). Many researcher employed phononic crystals with resonators’ body to improved quality of resonators, such as square shaped lattice PnC with hole employ with a resonator [26] gives quality 90,000, optimally shaped MEMS resonator [27] gives the quality 105,000, Cross shaped PnC MEMS resonator [23] gives 221,536, and gallium arsenide resonator [28] gives the quality of 6 million. 

This work describes a design of novel SR (Square-Rectangle) hole shaped Phononic crystal, and this phononic crystal applied to novel annular concentric rings shaped Aluminium Nitride piezoelectric MEMS resonator. 

At first, we have designed and simulated the unique silicon phononic crystal (PnC) structure of width 10 µm. we have also observed that reducing the value of filling fraction closer the band gaps. At second we have observed the band gaps generated by the 1D phononic crystal, and Eigen mode shapes of first 12 frequency bands. At third, we have observed the band gaps generated by the 2D phononic crystal, and Eigen mode shapes of first 12 frequency bands. At fourth, we have calculated transmission spectra through PnC for analyzing the band gaps, and leakage of energy. At fifth, we have designed and simulated the novel annular splits rings resonators with supporting tethers of width 4 µm to find the quality factor and investigated the displacement pattern and axes displacement of resonator, we have applied one dimensional phononic crystal (1D PnC) strips, and two dimensional phononic crystal (2D PnC) strips to annular splits rings resonators for further reduction of energy leakage and enhancement of the quality *Q* of resonator. In the end, we have investigated (*x*, *y*, *z*) displacement of resonator through FEA (Finite Element Analysis) simulation.

## 2. Phononic Crystals Structure and Band Gaps

During the last few years, Phononic crystal (PnC) is being under discussion of researchers due to its potential application in the field of technologies [15,24,25,29,30,31]. As the phononic crystals (PnCs) play an important role in development of micro/nanoelectronics, but it can also be involved in the improvement of quality of MEMS resonators. Many researchers employed phononic crystals to reduced anchor loss and improve the quality of resonators [19,26,32,33]. Dep et al. [34] also concluded that PnC based MEMS resonators can give the high quality. To reduce the anchor loss, one dimensional PnC strips were employed as an anchor of resonators [19]. Feng et al. [19] concluded that PnC strips can reduce the energy dissipated through anchors, and quality of ring resonators can be increased by increasing the number of phononic strips. 

Phononic crystals can prevent energy leakage from resonators and control the elastic wave (acoustic wave) propagation inside the band [35,36]. It means that Phononic crystal does not allow elastic wave (acoustic wave) propagation in their structure due to the presence of band gaps. For the first time the propagation of elastic waves in phononic crystals is govern by one dimensional Bloch waves [37]. Actually, in any materials, there is band structure, and this structure is understandable by band gaps. At certain frequencies, the propagation of wave occurs, which are called bands, but at some frequencies range the propagation of wave stopped (cause of mechanical waves inside the PnC) are called band gaps. Phononic crystal can be one dimensional(1D) [19,38,39], two dimensional (2D) [35,40], and three dimensional(3D) [41,42]. To deduct the wider bandgap different shapes of phononic crystals were designed such as cylindrical pillars [43], honey comb [44], holes [30], square plates [39], etc.

## 3. Analysis of Phononic Band Gaps

**Figure 1:**
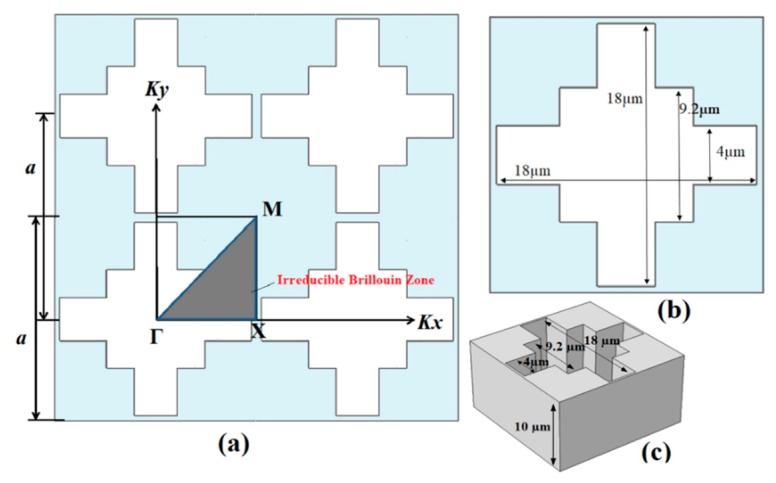
(**a**) Irreducible Brillouin Zone of phononic crystal. (**b**) Schematic of unit cell with lattice parameter *a* = 18.6 µm. (**c**) 3D view of PnC unit cell.

The band gap in Phononic crystal (PnC) depends upon the properties of materials from which PnC made, density, and speed of sound in materials. Simply, we can say that band gap’s position with respect to frequency is the ratio of sound velocity to the size of periodic unit cell, and filling factor [45,46]. We have investigated the wave propagation in a novel air SR (Square-Rectangle) shaped phononic crystal unit cell consist of Si (silicon, anisotropic) through FE parametric solver which can sweep wave vector *k*. The air SR shape means the holey square and holey rectangles in PnC unit cell. The geometrical dimension of unit cell is 18.6 µm × 18.6 µm with centered horizontal and centred vertical air rectangles 18 µm × 4 µm (rectangles hole) and centered air square of 9.2 µm (square hole) each side, as shown in Figure 1b. The dimensions of centered rectangles hole and centered square hole, as shown in Figure 1b. The 3D view of PnC structure as shown in Figure 1c.

We analyzed the above periodic unit cell to evaluate the frequency response for 1D phononic crystal. The *k* parametric sweep fluctuates from 0 to 1. Where (0–1) indicate wave number of irreducible Brillouin zone (Γ–Χ). More generally, *k* swept from Γ to Χ (0 to π*/a*).

Moreover, the dispersion occurs when different wavelengths’ waves have different velocities of propagation. The dispersion relation associated with wave vector *k* to its frequency as follows

(1) $$k=\frac{\omega }{c}$$

where ‘*ꞷ*’ and ‘*c*’ are the angular frequency and wave velocity respectively.

**Figure 2:**
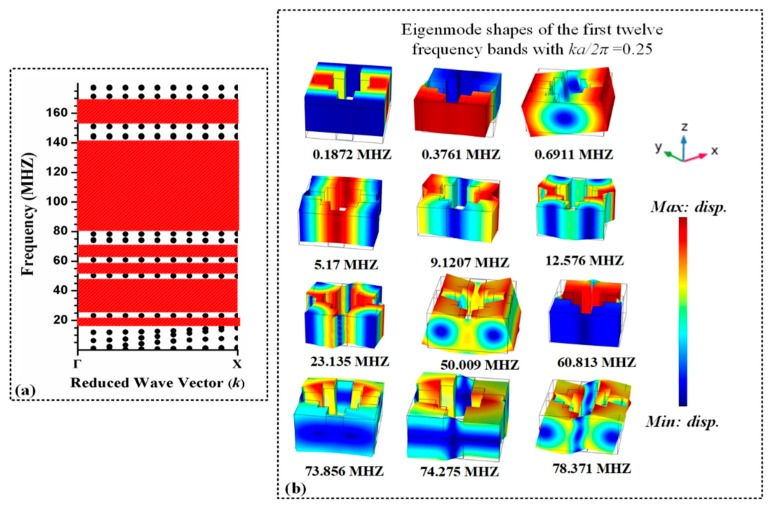
(**a**) Frequency band structure curves in irreducible Brillouin zone [(0–1) (Γ–Χ)] of 1D PnC investigated through FE method. (dotted lines indicate the dispersion curves of elastic waves) red area represent complete bandgaps. (**b**) Eigen mode shapes of the first 12 frequency bands.

Particularly for phononic crystal the dispersion relation is complex and is represent by frequency band curves schematic as shown in Figure 2.

Figure 2a obtained from FEA (Finite Element Analysis) simulation, red region shows that there is no wave propagation or no resonant mode in structure which covers a wide and complete bandgap. The band gaps generated by the 1D phononic crystal in Brillouin zone (Γ–Χ) is depicted in Figure 2a.

It is worth mention that bandgap also depend upon the geometrical dimension, and inclusion filling factor in PnC by using the following relation

(2) $${\left(f_{\alpha }\right)}_{Si}=\frac{\mathrm{area}\;\mathrm{of}\;\mathrm{inclusion}\;}{\mathrm{area}\;\mathrm{of}\;\mathrm{unit}\;\mathrm{cell}}$$

Air is used as high acoustic impedance material, and Aluminum Nitride (AIN) is low acoustic impedance material. The filling fraction of this SR (square rectangles) hole PnC is 0.542.

If the horizontal and vertical air rectangle is taken as 17 µm × 5 µm in size, and centered air square of 9 µm of each side, the band gap has reduced. We have observed that reducing the value of filling fraction closer the band gaps. The band gaps created into higher order modes due to smaller air inclusion area. The filling fractions and the band gap of phononic crystal obtained from different geometrical dimension, as shown in Table 1

We have also analyzed the above periodic unit cell to evaluate the frequency response for 2D phononic crystal. In 2D PnC structure the *k* parametric sweep fluctuates from 0 to 3. Where (0–1), (1–2), and (2–3) indicate wave number of irreducible Brillouin zone (Γ–Χ), (Χ–Μ), and (Μ–Γ) respectively. More generally *k* swept from Γ to Χ (0 to π*/a*), Χ to Μ (0 to π*/a*), and then Μ to Γ (π*/a* to 0).

**Figure 3:**
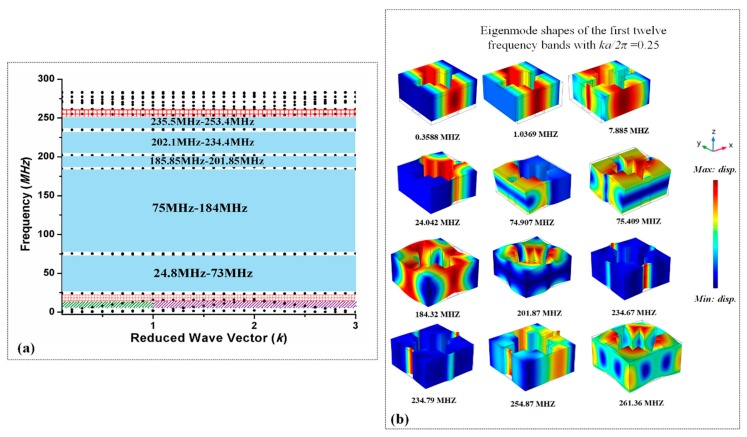
(**a**) Frequency band structure curves in irreducible Brillouin zone [(Γ–Χ), (Χ–Μ), and (Μ–Γ)] of 2D PnC investigated through FE method. (dotted lines indicate the dispersion curves of elastic waves) blue area represent complete bandgaps. (**b**) Eigen mode shapes of first 12 frequency bands.

Figure 3 obtained from FEA (Finite Element Analysis)) simulation, blue region shows that there is no wave propagation or no resonant mode in structure, which covers a wide and complete bandgap.

The red shaded, green shaded, and purple shaded area represents the deaf acoustic mode or deaf bandgap [47,48], because only resonant modes are allow to propagate in these range of frequencies. Deaf bands are the uncoupled band in dispersion relation. The frequency band structure of 3D plate model and eigenmode shapes of frequency bands with *ka/2*π = 0.25 is shown in Figure 3.

As this PnC structure employed to resonator for further reduction of energy leakage, so we have calculated transmission spectra through PnC for analyzing the leakage of energy, because transmission of acoustic waves is forbidden in a bandgap. The transmission is defined as the ratio of transmitted waves to incident waves, and calculated as the difference of probe displacement and source displacement.

(3) $$T=10\log\left(\frac{\;d_{\mathrm{probe}\;}^{2}}{d_{\mathrm{source}}^{2}}\right)$$

**Figure 4:**
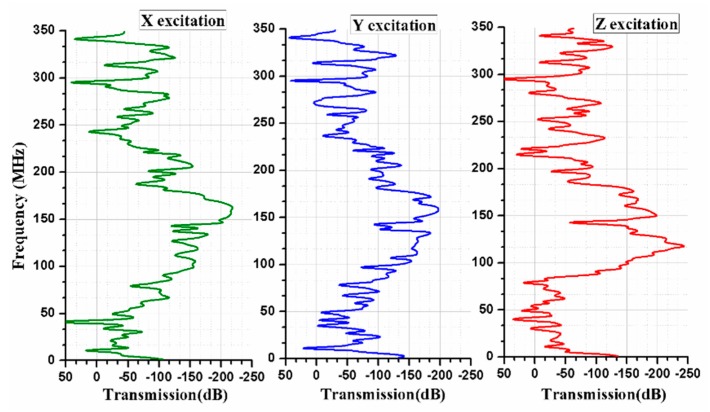
Transmission Vs Frequency for three different excitations investigated through the FE method.

As the phononic crystal is employed to resonator with supporting tether length 12 µm, and width 4 µm on both sides of resonator. The motion of resonator is in *y-*direction and movement of Poisson coupling in *x-*, and *z-*directions. The *x, y,* and *z* excitations, as shown in Figure 4.

**Figure 5:**
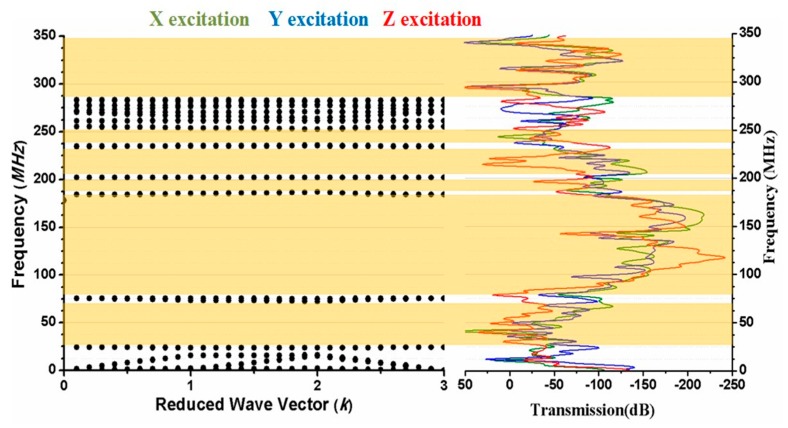
Comparison between bandgap graph and transmission for 2D PnC with lattice *a* = 18.6 µm, *f* = 0.542.

We have calculated the transmission wave spectra through finite thickness of 10 µm Phononic crystals as shown in Figure 4. Here we see that the attenuation peak is wider for *x* and *y* excitation as well as for *z* excitation, and no dip in transmission as compared in band diagram of Figure 5. A very minor transmission dip is observed only at 120 MHz in *z* excitation. Form Figure 5 we see that the band gap, wide peak attenuation, and operational frequency of resonator lie in the same frequency region. Thus, wider peak of attenuation makes a broader choice of desire operational frequencies of resonators. Thus, it is clear from Figure 5 that the transmission is very low and acoustic wave is banned in the yellow highlighted area (band gaps area).

## 4. Device Analysis

**Figure 6:**
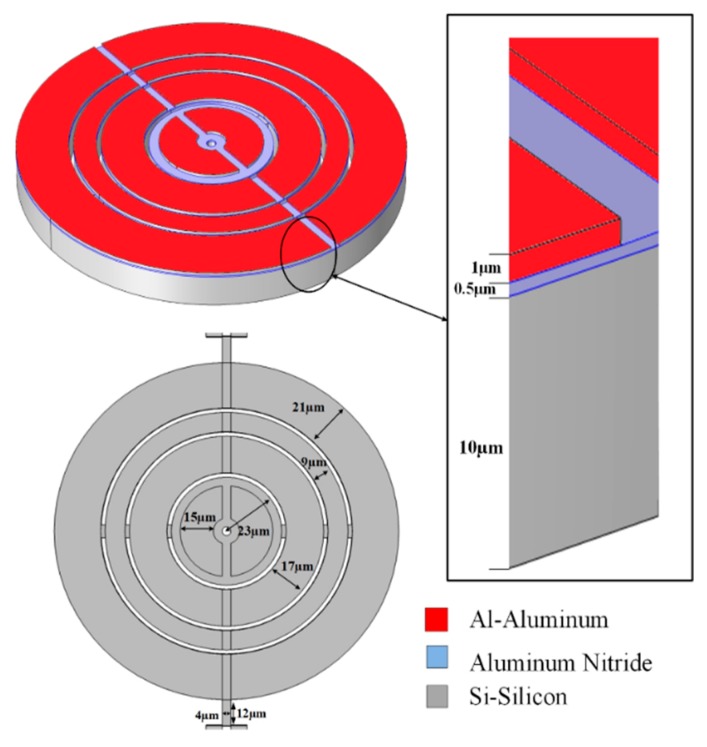
Schematic of annular concentric rings shaped Aluminium Nitride piezoelectric resonator with tether width 4 µm.

Annular concentric rings shaped Aluminum Nitride piezoelectric MEMS resonator is designed in this work, as shown in Figure 6.

The resonator is described with width of tether (*T_w_* = 4 µm) without phononic crystal (PnC), and also with one dimensional phononic crystal (1D PnC), and two dimensional phononic crystal (2D PnC).

The equation of frequency for annular ring, disks [8,9,45,46,47,49,50] and mode shape [51] with general elastic boundary conditions is given in the following way.

(4) $$\left[\begin{array}{cc} J_{1}\left(\frac{\omega R_{0}}{c}\right)\left[M_{1}\left(\frac{\omega R_{0}}{c}\right)-\left(1-\sigma \right)\right] & Y_{1}\left(\frac{\omega R_{0}}{c}\right)\left[N_{1}\left(\frac{\omega R_{0}}{c}\right)-\left(1-\sigma \right)\right]\\ J_{1}\left(\frac{\omega R_{i}}{c}\right)\left[M_{1}\left(\frac{\omega R_{i}}{c}\right)-\left(1-\sigma \right)\right] & Y_{1}\left(\frac{\omega R_{i}}{c}\right)\left[N_{1}\left(\frac{\omega R_{i}}{c}\right)-\left(1-\sigma \right)\right] \end{array}\right]$$

where (*J*, and *Y*) are the first, and second kind of Bessel functions, and (*M*, and *N*) are modified form of Bessel functions, *R*_0_, and *R_i_* are the outer and inner radius.

The Aluminum Nitride (AlN) as piezoelectric material is taken in ring shaped resonator, so the resonant frequency [9,52] can be governed in the following way

(5) $$f=\frac{1}{\lambda }\sqrt{\frac{E_{p}}{\rho \left(1-\sigma^{2}\right)}}$$

where *λ =* 2*W*, *E_p_*, *ρ*, and *σ* are width of resonator mode (wavelength), Young’s Modulus, Poisson’s ratio, and effective mass density of piezoelectric material, respectively. We have set the primary alignment of resonators structure to <110> frame of reference for silicon elasticity matrix [53] in (COMSOL Multiphysics 5.4) as below

$$\left[\;\begin{array}{cccccc} 194.5 & 35.7 & 64.1 & 0 & 0 & 0\\ 35.7 & 194.5 & 64.1 & 0 & 0 & 0\\ 64.1 & 64.1 & 165.7 & 0 & 0 & 0\\ 0 & 0 & 0 & 79.6 & 0 & 0\\ 0 & 0 & 0 & 0 & 79.6 & 0\\ 0 & 0 & 0 & 0 & 0 & 50.9 \end{array}\right]\mathrm{Gpa}$$

Some other important parameters of medium/materials used in this work are listed in Table 2.

**Figure 7:**
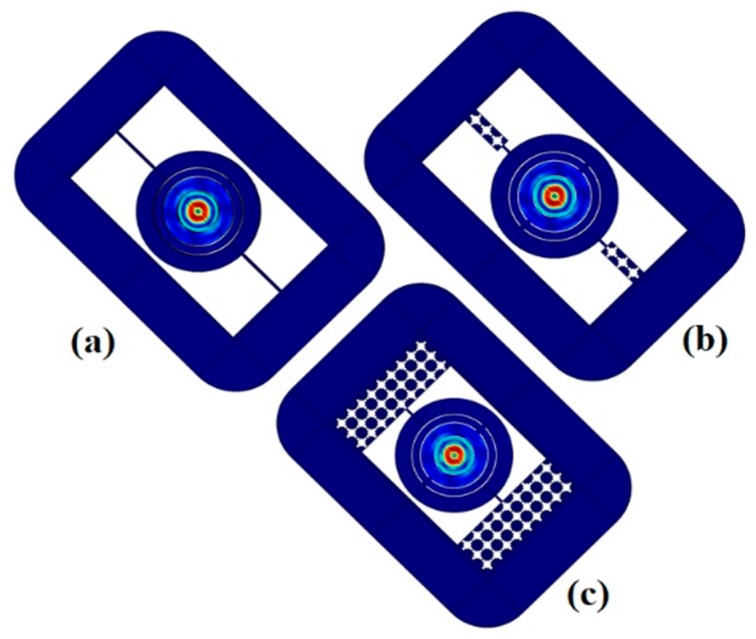
FEA Simulations of Displacement field pattern in the resonators with perfect match layer (PML) width = 1.5*λ*: (**a**) (Without Pnc) Mode shape at frequency *f* = 188.55 MHz. (**b**) (With 1D Pnc) Mode shape at frequency *f* = 188.6 MHz. (**c**) (With 2D Pnc) Mode shape at frequency *f* = 188.6 MHz.

**Figure 8:**
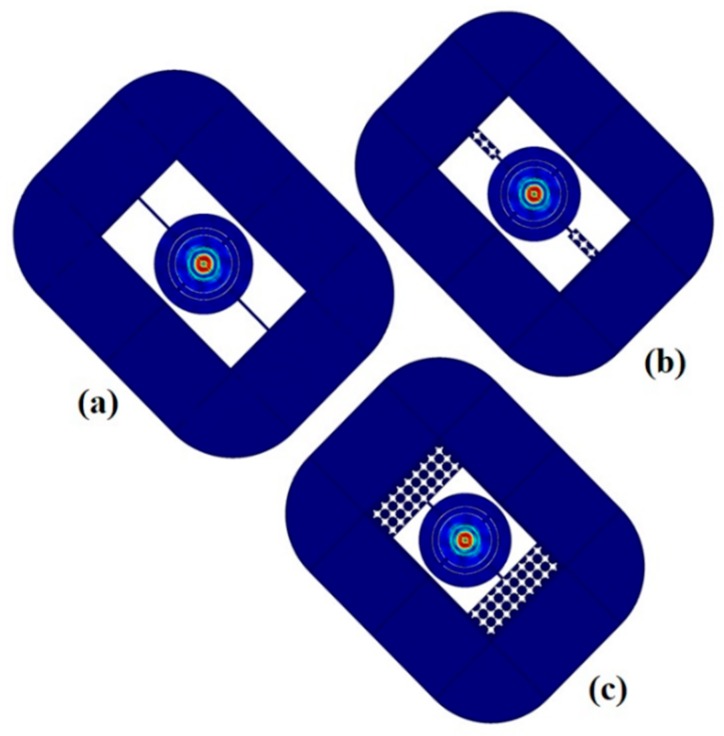
FEA Simulations of Displacement field pattern in the resonators with perfect match layer (PML) width = 3*λ*: (**a**) (Without Pnc) Mode shape at frequency *f* = 188.57 MHz. (**b**) (With 1D Pnc) Mode shape at frequency *f* = 188.62 MHz. (**c**) (With 2D Pnc) Mode shape at frequency *f* = 188.6 MHz.

When the potential is applied between aluminum electrodes, the electric field is developed the induced mechanical strain along aluminum nitride piezoelectric material, this is due to inverse effect of piezoelectric, as a result mode of vibration appears. The mode shape vibration of resonator is depicted in Figure 7 and Figure 8. Both Figures indicate the good pattern of mode shapes based upon the analysis of corresponding eigen frequencies through FEA simulation. 

In this study we have also obtained high *Q* by employing PnCs to resonators through FEA (Finite Element Analysis) simulation. There are few energy loss mechanisms comprise the total Q-factor of the device [54]. Their losses are given as Support (anchor) loss, Viscous damping, Electrical damping, Loss due to structural asymmetry, and Thermo-Elastic Damping (TED).

(6) $$\frac{1}{Q_{\mathrm{total}}}=\frac{1}{Q_{\mathrm{anchor}}}+\frac{1}{Q_{\mathrm{viscous}\;\mathrm{damping}}}+\frac{1}{Q_{\mathrm{electrical}\;\mathrm{damping}}}+\frac{1}{Q_{\mathrm{material}}}+\frac{1}{Q_{\mathrm{TED}}}$$

In this study only anchor loss is considered (through FEA simulation), and the other losses are completely omitted. Due to nature of motion resonator it is not affected by air damping, and the material losses are difficult to predict, so the major loss is anchor loss [33,55,56]. The energy loss mechanism in MEM resonator is also demonstrated by Akhieser effect (AKE) [57]. Moreover Frangi et al. [58,59] reported that all thermoelastic losses (Akhieser losses) are included due to sources of dissipation. These Akhieser losses are generally negligible because piezoelectric MEM resonators operate in one of its bulk modes, and only the anchor loss deserve attention. 

We have analyzed the Quality *Q* of proposed resonator without PnC, employed with 1D PnC, and with 2D PnC by the following summarized relation [60,61].

(7) $$Q=2\pi \frac{E_{s}}{E_{l}}$$

where *E_s_* and *E_l_* are the energy stored (vibration energy) and energy loss (energy dissipation per cycle of vibration) in resonator.

**Figure 9:**
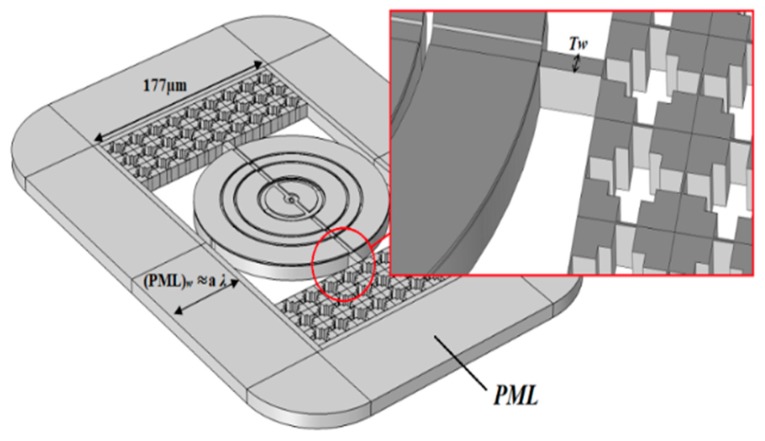
Schematic view of concentric rings resonator using phonic crystals surrounded by perfect match layers (PML) (inset: closed view of tether attached with 2D PnC.

Figure 9 show that the tether (width 4 µm) is attached with 2D PnC, and resonator is bounded with PML (perfect match layer). The width of PML is set as varying ‘a*λ*’ (*a* = 1.5 and 3, and *λ* = 45 µm) (Table 3). Elastic waves are produced by resonator through tether, this is the major loss of energy. Specific dimension of PML can prevent resonator from reflecting elastic waves, because the elastic waves can be absorbed by PML [57,62,63]. Thus, we have used the perfect match layer through FEA (Finite Element Analysis) simulation to reduce the energy leakage through tethers. Reducing the energy leakage means increasing the quality factor. So *Q*_anchor_ is obtained by resonant frequency *f*_0_ over band width −3 dB [33,56,58]

(8) $$Q_{\mathrm{anchor}}=\frac{f_{0}}{-3\;\mathrm{dB}\left(\Delta f\right)}=\frac{Re\left(\omega \right)}{2Im\left(\omega \right)}$$

where ‘*ꞷ’* is the desired mode’s eigen frequency of the resonator in FEA simulation.

**Figure 10:**
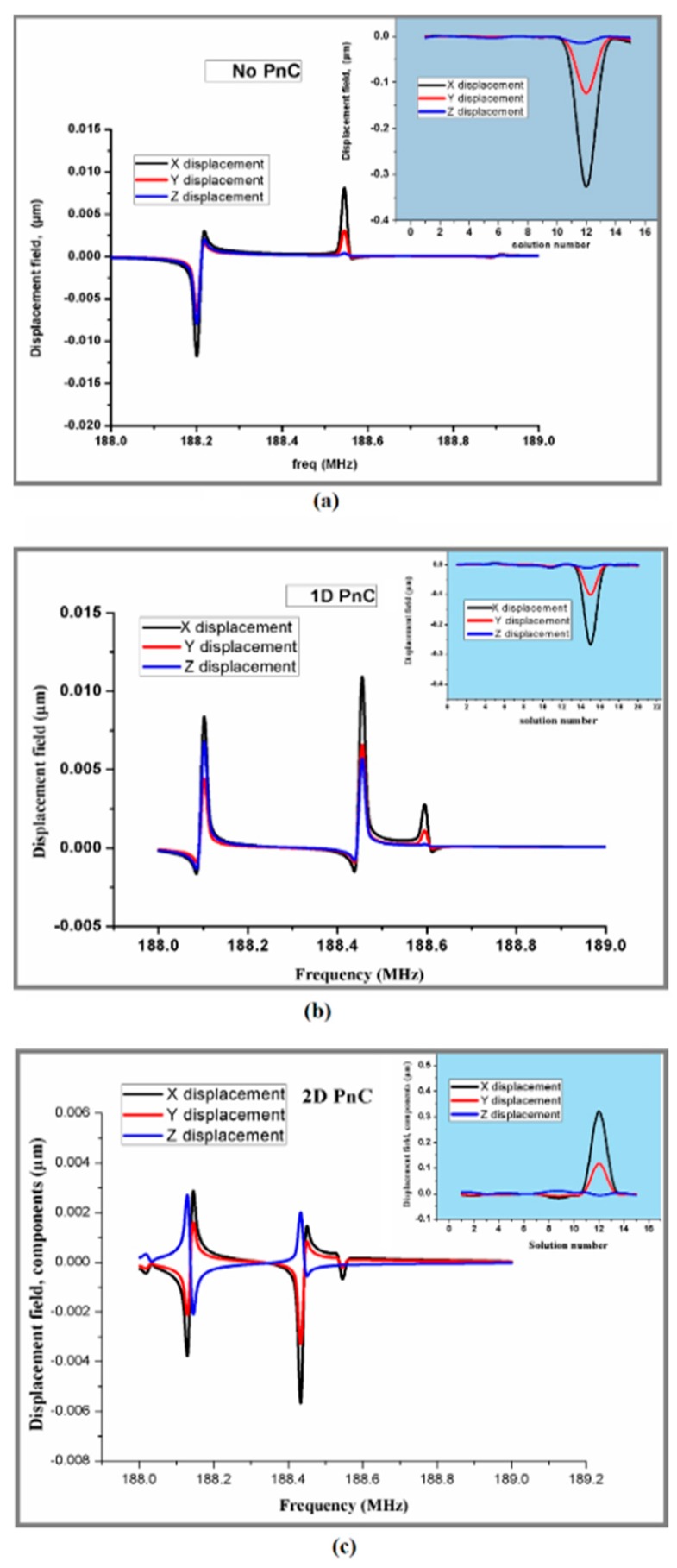
Investigation of (*x*,*y*,*z*) displacement of resonator: (**a**) without PnC structure.(**b**) 1D PnC structure. (**c**) 2D PnC with perfect match layer (PML) width = 1.5*λ* obtained from FEA simulations.

**Figure 11:**
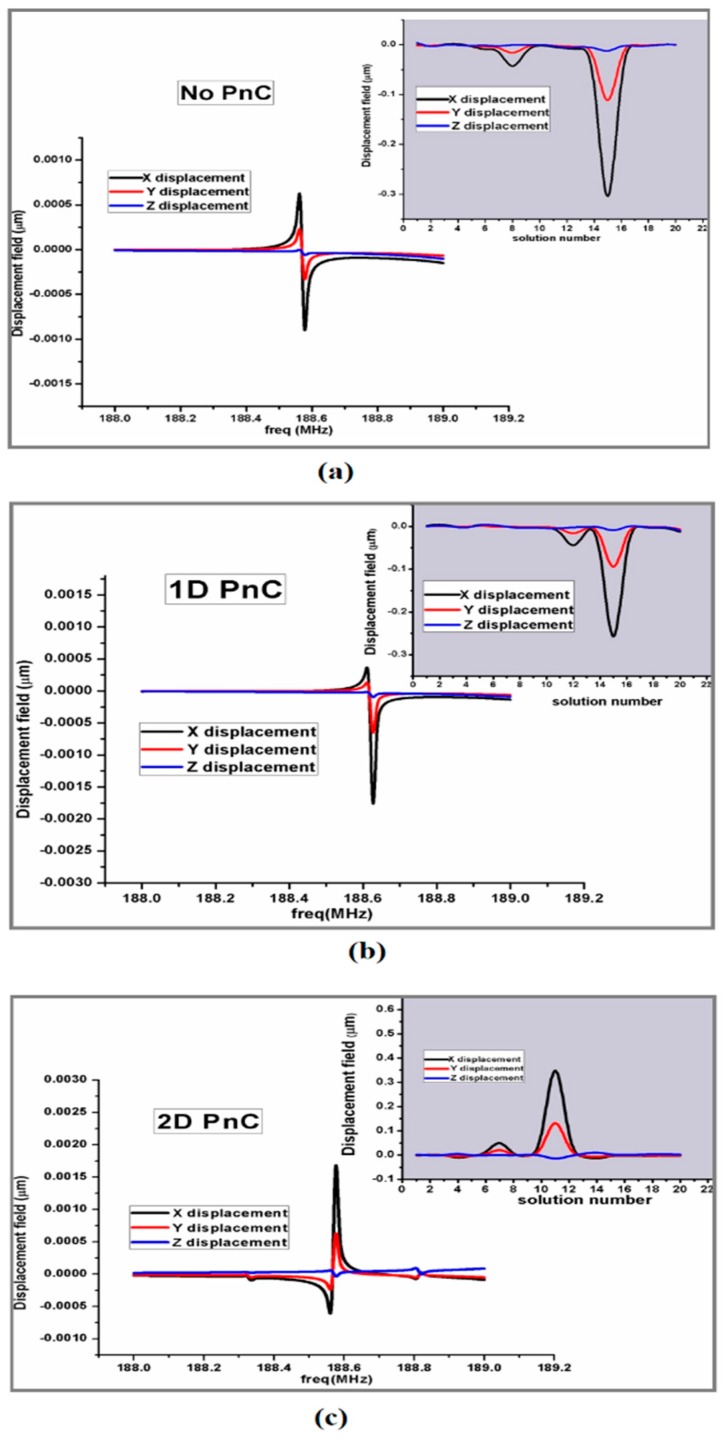
Investigation of (*x*,*y*,*z*) displacement of resonator: (**a**) Without PnC structure. (**b**) 1D PnC structure. (**c**) 2D PnC with perfect match layer (PML) width = 3*λ* obtained from FEA simulations.

The response of annular rings resonators is obtained by suppling the voltage source of 1 V to electrodes. Figure 10 and Figure 11 show the displacement of resonators along *x*, *y*, and *z* directions without, and with pnC structure through frequency domain FEA simulations. 

These results declare that resonators with perfect match layer (PML) width approximately equal to 3*λ* ≈ 135 µm reduced the displacement of vibration, as shown in Figure 11. The Perfect match layer (artificial absorbing layers) technique is firstly described by Pierre [64]. Moreover, wide PML curtail the frequency domain and acts as an isotropic absorber or simply we can say it acts like an artificial boundary. 

**Figure 12:**
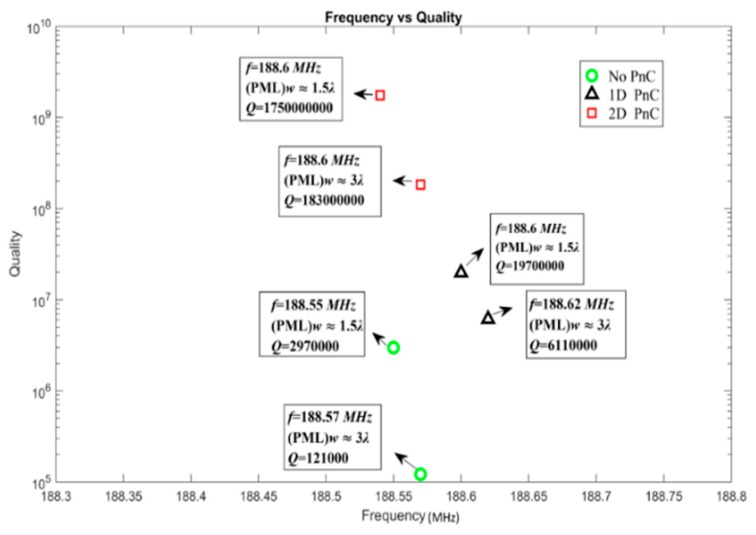
Quality of resonators without PnC structure, with 1D PnC structure, with 2D PnC (with perfect match layer (PML) width = 1.5*λ*, 3*λ*) obtained from FEA simulations.

Figure 12 differentiate the Quality (obtained from FEA simulations) between resonators without PnC structure, with 1D PnC structure, with 2D PnC (with perfect match layer (PML) width = 1.5*λ*, 3*λ,* respectively). These results show that Quality *Q* of resonator is the highest when 2D Phononic crystal structure is employed to resonators.

We see that, this unique design of resonators gives high quality, and when PnC is employed to resonator, the energy isolate from the resonator to substrate (reducing the loss of energy) as a result *Q* further increased. Furthermore, band gap of designed PnC covers the operating frequency of designed resonators, and the design of this SR PnC will help to increase the quality of further designing of resonators in future.

## 5. Conclusions

In this work, we have presented a new approach of annular concentric split rings microelectromechanical resonators with tether configuration to reduce anchor loss and gives very high-quality factor (*Q*) 2.9 Million. These resonators were operating between the frequency 188.55 MHz and 188.62 MHz. In this works we have also proposed SR (square rectangle) hole shaped phononic crystal structure, which gives very wide and complete band gaps. When SR (square rectangle) shaped one dimensional phononic crystal (1D PnC), and two dimensional phononic crystal (2D PnC) is employed to novel design rings MEMS resonators the quality factor (Q) further improved to 19.7 Million and 1750 Million, respectively. We have concluded that value of filling fraction is reduced then band gaps become closer. It is observed that wider peak of attenuation makes broader desired operational frequencies of resonators.

We have also verified the harmonic response of annular rings resonators by perfect match layers (PML) technique surrounded to resonators with varying width 1.5*λ*, and 3*λ* effectively reduce the vibration displacement.

## Figures and Tables

**Table 1:** Simulation parameters of PnC structures.

Rectangle Hole (µm^2^)	Square Hole (µm^2^)	Maximum Bandgap (MHz)	(*f_α_*)*_si_*
18 × 4	9.2 × 9.2	75–184	0.542
17 × 5	9 × 9	103.8–195.5	0.511
16 × 6	8.8 × 8.8	121–192.5	0.481

**Table 2:** Properties of Materials/Medium.

Medium/Materials	Parameters
Silicon (Si)	Density (*ρ*) = 2330 kg/m^3^
Aluminum Nitride (AIN)	Density (*ρ*) = 3300 kg/m^3^ Relative permittivity (*ε*) = 9 Poisson’s ratio (*ν*) = 0.24 Young’s Modulus (*E*) = 320 Gpa
Aluminum (Al)	Density (*ρ*) = 2700 kg/m^3^ Young’s Modulus (*E*) = 70 Gpa Poisson’s ratio (*ν*) = 0.35 Electrical conductivity (*σ*) = 35.5 × 10^6^ S/m Coefficient of thermal expansion (*α*) = 23.1 × 10^−6^/K Heat capacity (*Cp*) = 904 J/Kg K Thermal conductivity (*κ*) = 237 W/mK

**Table 3:** Simulation parameters of PML width surrounded with resonators.

Structure	Frequency (MHz)	PML Width (λ ≈ 45 µm)	Quality
Without PnC	188.55	1.5*λ*	2970,000
	188.57	3*λ*	121,000
With 1D PnC	188.6	1.5*λ*	19,700,000
	188.62	3*λ*	6,110,000
With 2D PnC	188.6	1.5*λ*	1,750,000,000
	188.6	3*λ*	183,000,000
